# Narrow-wide row planting pattern improves the light environment and seed yields of intercrop species in relay intercropping system

**DOI:** 10.1371/journal.pone.0212885

**Published:** 2019-02-26

**Authors:** Lingyang Feng, Muhammad Ali Raza, Yuankai Chen, Muhammad Hayder Bin Khalid, Tehseen Ahmad Meraj, Faiza Ahsan, Yuanfang Fan, Junbo Du, Xiaoling Wu, Chun Song, Chuanyan Liu, George Bawa, Zhongwei Zhang, Shu Yuan, Feng Yang, Wenyu Yang

**Affiliations:** 1 College of Agronomy, Sichuan Agricultural University, Chengdu, Sichuan, China; 2 Key Laboratory of Crop Eco-physiology and Farming System in Southwest, Ministry of Agriculture, Chengdu, Sichuan, P.R. China; 3 Maize Research Institute, Sichuan Agricultural University, Chengdu, Sichuan, PR China; 4 College of Resources, Sichuan Agricultural University, Chengdu, Sichuan, China; Wageningen University, NETHERLANDS

## Abstract

Different planting patterns affect the light interception of intercrops under intercropping conditions. Here we revealed that narrow-wide-row relay-intercropping improves the light interception across maize leaves in wide rows (60cm) and narrow rows (40cm), accelerated the biomass production of intercrop-species and compensated the slight maize yield loss by considerably increasing the soybean yield. In a two-year experiment, maize was planted with soybean in different planting patterns (1M1S, 50:50cm and 2M2S, 40:60cm) of relay-intercropping, both planting patterns were compared with sole cropping of maize (M) and soybean (S). As compared to M and 1M1S, 2M2S increased the total light interception of maize leaves in wide rows (WR) by 27% and 23%, 20% and 10%, 16% and 9% which in turn significantly enhanced the photosynthetic rate of WR maize leaves by 7% and 5%, 12% and 9%, and 19% and 4%, at tasseling, grain-filling and maturity stage of maize, respectively. Similarly, the light transmittance at soybean canopy increased by 218%, 160% and 172% at V_2_, V_5_ and R_1_ stage in 2M2S compared with 1M1S. The improved light environment at soybean canopy in 2M2S considerably enhanced the mean biomass accumulation, and allocation to stem and leaves of soybean by 168%, and 131% and 207%, respectively, while it decreased the mean biomass accumulation, and distribution to stem, leaves and seed of maize by 4%, and 4%, 6% and 5%, respectively than 1M1S. Compared to 1M1S, 2M2S also increased the CR values of soybean (by 157%) but decreased the CR values of maize (by 61%). Overall, under 2M2S, relay-cropped maize and soybean produced 94% and 69% of the sole cropping yield, and the 2M2S achieved LER of 1.7 with net income of 1387.7 US $ ha^-1^ in 2016 and 1434.4 US $ ha^-1^ in 2017. Our findings implied that selection of optimum planting pattern (2M2S) may increase the light interception and influence the light distribution between maize and soybean rows under relay-intercropping conditions which will significantly increase the intercrops productivity. Therefore, more attention should be paid to the light environment when considering the sustainability of maize-soybean relay-intercropping via appropriate planting pattern selection.

## Introduction

In China, intensive farming has been practiced with high inputs of chemicals, fertilizers, seeds, and irrigation, due to the high food security pressure. This situation has raised serious environmental problems [1], including groundwater pollution by leaching of nitrogen from soil layers [2], acidification of soil [3] and emission of harmful gases to air [4]. The nitrogen loss during maize planting is an especial concern [5]. To guarantee both food production and environmental security, we have to adopt best agronomic practices such as appropriate planting systems which have the ability to use sunlight and land resources efficiently with minimum inputs, for instance, intercropping and relay intercropping systems [6,7]. Relay intercropping is one of the important agronomic practices to increase seed yield [8,9]. However, as compared to intercropping systems, the advantage of relay-intercropping system is higher because in intercropping systems both crops almost have the similar growth periods and they required high amount of inputs to produce higher intercrop yields, whereas under relay intercropping system both crop species have different growth periods and have complementary resource use in time [7,8,10,11]. In addition, for maize soybean relay-intercropping system, the land equivalent ratio (LER, described as the relative farmland that is needed for sole crops to produce similar crop yields as intercrops) often reaches 1.7–1.8 when both crops planted at their optimal planting density, which increases its popularity among farmers, especially among small farmers [10]. However, during the co-growth period per plant growth rates decrease and competition for sunlight, nutrients and land are exacerbated between maize and soybean under maize soybean relay intercropping system [11,12]. The reduced light intensity perceived by soybean plants promoted by maize canopy not only decreased the seed yield [13] but also lower the seed quality of soybean [14].

Planting pattern in intercropping systems changed the micro-climate, especially the light conditions of intercrops [8]. The adjacent growing of crops always cause mutual shading among individual intercropped plants [15]. Previously, it has been reported that upper canopy leaves shade middle strata leaves in maize and mutual shading of leaves reduces the photosynthetic capacity of maize plants which changes the crop morphology, electron transport chain in photosynthetic process and concentrations of enzymes related to carbohydrate assimilation [16,17]. Importantly, the major part of assimilates for seed filling process is obtained from the current carbohydrate production of maize leaves after tasseling, and subsequent translocation to the seeds [18,19]. Similarly, under this system, maize shading significantly affected the light environment of soybean canopy in terms of light quality and quantity [8]. The soybean is extremely sensitive to shading conditions [20] and soybean plants suffer from maize shading during their co-growth period under relay intercropping systems [21]. This shading environment inhibits the leaf growth and enlargement by controlling the cell proliferation of mesophyll cells in soybean [22]. Scientist have also confirmed that the stem diameter, root biomass, and plant biomass decrease under shading conditions that ultimately decrease the seed yield of soybean in relay intercropping system [8,11]. In addition, the shading conditions under relay intercropping systems considerably decrease the rate of sucrose transportation and stem breaking strength of soybean plants [23]. Therefore, by selecting the appropriate genotypes and planting pattern we can increase crop yield and quality under prevailing conditions [24,25,26].

**Fig 1:**
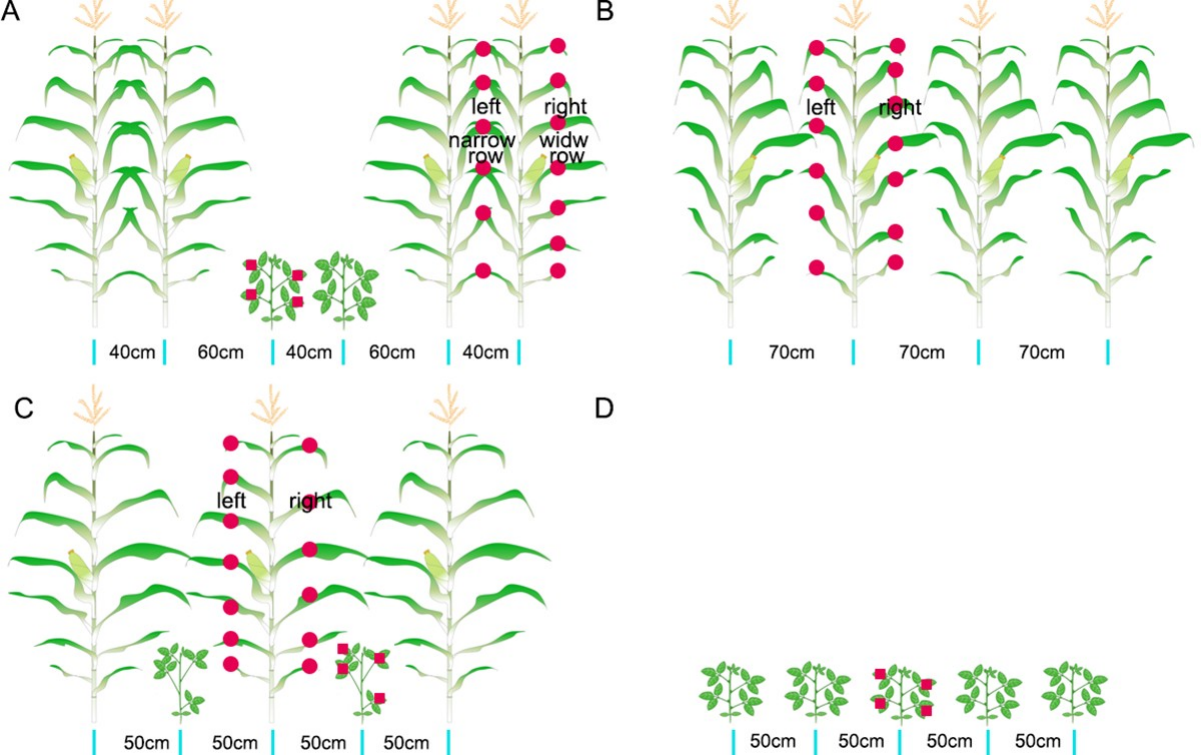
Schematic representation of different maize-soybean planting patterns. A, B, C and D represent the general layout of 2M2S (40 cm + 160 cm; maize narrow-wide row planting, i.e., the relay intercropping combination of two crop strips with a total width of 200 cm, consisting of two rows of maize and two rows of soybean with 40-cm row width for maize and soybean, and 60-cm spacing between the adjacent rows of maize and soybean), 1M1S (100 cm; with width of one meter strip, i.e., one row of maize and one row of soybean with 50-cm spacing between the adjacent rows of maize and soybean), M (70 cm; with equal row configuration in one strip for maize row to maize row arrangement) and S (50 cm; with equal row configuration in one strip for soybean row to soybean row arrangement), respectively. 2M2S and 1M1S are intercropping system, M and S are sole cropping system of maize and soybean, respectively. The red film placed at the middle of maize and soybean leaves for one day from 6:00 am to 20:00 pm was used for the measurement PPFD as shown in the figure.

In China, maize soybean relay-intercropping system follows the two main planting patterns: (i) modern narrow-wide row relay-intercropping; “40 cm: 60 cm: 40 cm: 60 cm” maize narrow-wide row planting, i.e., relay-intercropping combination of 2 crop strips with a total width of 200cm, consisting of 2 rows of soybean and 2 rows of maize with 40-cm row width (narrow) for soybean and maize, and 60-cm spacing (wide) between the rows of soybean and maize **Fig 1A** [7,11,27], and (ii) traditional row relay-intercropping; “50 cm + 50 cm” maize soybean equal row planting, i.e., 1 row of soybean and 1 row of maize with 50-cm spacing between the rows of soybean and maize **Fig 1B** [21]. Therefore, it is important to investigate the effects of different planting patterns on light interception and distribution in maize and soybean plants under relay intercropping system. In past studies, scientists have mainly focused on the morphological and photosynthetic characteristics of intercrop species under relay intercropping conditions [8,11]. However, no study has been carried out to investigate the light interception and distribution pattern in maize and soybean plants under relay intercropping conditions. Thus, a comprehensive study was required to understand the light environment of maize and soybean under the maize soybean relay intercropping system. Therefore, a two-year field experiment was carried out to investigate the photosynthetically active radiation (PAR) distribution among maize and soybean plants under different planting patterns. Main objectives of the present experiment were (i) to quantify the total interception of PAR at maize plants under different planting patterns; (ii) to analyze how different planting patterns affect the PAR transmittance at soybean canopy; (iii) to investigate how the variations of PAR transmittance affect the biomass accumulation of maize and soybean at different growth stages and grain yields under different planting patterns.

## Materials and methods

### Ethics statement

No specific permissions were needed for these field experiments. All experiments were performed according to institutional guidelines of Sichuan Agricultural University, China.

### Experimental location

The experiments were carried out in 2016 and 2017 at Renshou Research Farm of Sichuan Agricultural University, Sichuan Province, China (N30°16 '4', E104°12'53 ", altitude 482 m asl). The climate of the study area was humid and subtropical and it has the annual average temperature of 17.4°C, annual average rainfall 1009.4 mm, annual average sunshine 1196.6 h and a frost-free period of 312 days. Weather data during the growing seasons from 2016 to 2017 includes monthly rainfall, average temperature, humidity and wind speed (**Table 1)**. Total rainfall in 2017 was less than 2016, irrigation was applied at the time of soybean sowing and fifth trifoliate stage of soybean. The soil has a purple clay texture with 6.8 pH, 13.6 g kg^−1^ organic matter, 0.43 g kg^−1^ total N, 0.36 g kg^−1^ total P, 7.16 g kg^−1^ total K, 52.9 mg kg^−1^ available N, 10.8 mg kg^−1^ available P, and 107.8 mg kg^−1^ available K in the 0–20 cm soil layer.

**Table 1:** Monthly rainfall, average temperature, humidity, and wind speed from March to October in the growing seasons of 2016 and 2017.

Month	Year							
	2016				2017			
	Rainfall (mm)	Average T (°C)	Humidity (%)	Wind Speed (ms^-1^)	Rainfall (mm)	Average T (°C)	Humidity (%)	Wind Speed (ms^-1^)
**March**	41.9	15.41	58.32	0.36	29.5	13.17	55.33	0.35
**April**	65.2	19.33	60.21	0.43	52	19.57	57.35	0.46
**May**	93.3	22.71	62.35	0.51	40.7	23.57	56.32	0.52
**June**	125.4	26.37	65.31	0.40	55.50	25.01	56.41	0.42
**July**	261.1	27.63	89.44	0.77	82.30	29.11	62.34	0.36
**August**	126.2	28.53	68.91	0.62	204.8	27.77	80.13	1.21
**September**	172.8	22.47	73.25	0.82	48.10	23.57	54.39	0.47
**October**	21.12	19.33	56.21	0.47	58.50	17.61	57.82	0.42
**March-October**	907.02	22.72	66.75	0.55	571.40	22.42	60.01	0.53

### Experimental design and treatments

The Chuandan-418 (semi-compact maize) and Nandou-12 (shade-tolerant soybean) maize and soybean varieties respectively, were selected for the experiments. Different planting patterns described as follows in **Fig 1**: (2M2S) “40 cm: 60 cm: 40 cm: 60 cm” maize narrow-wide row planting (modern planting pattern generally used for maize and soybean production under relay intercropping), i.e., the relay intercropping combination of two crop strips with a total width of 200 cm, consisting of two rows of maize and two rows of soybean with 40-cm row width for maize and soybean, and 60-cm spacing between the adjacent rows of maize and soybean [8,11,27]; (1M1S) “50 cm: 50 cm” with width of one meter strip (traditional row relay-intercropping), i.e., one row of maize and one row of soybean with 50-cm spacing between the adjacent rows of maize and soybean [21]; (M) “70 cm” with equal row configuration in one strip for maize row to maize row arrangement; (S) “50 cm” with equal row configuration in one strip for soybean row to soybean row arrangement. 2M2S and 1M1S are the intercropping systems, M and S are sole cropping system of maize and soybean, respectively.

The experiments were laid out using a randomized complete block design with three replicates. Every experimental block size was 36 m^2^ (6 m × 6 m) in the intercropping system, including six rows of maize and six rows of soybean. In sole cropping system, the size of each experimental block was 42 m^2^ (6 m × 7 m), consisting of 10 rows of maize and 14 rows of soybean in M and S, respectively. The maize crop was sown on 29^th^ of March 2016 and 5^th^ of April 2017 and harvested on 13^th^ of August 2016 and 16^th^ August 2017. Soybean was sown on 19^th^ June 2016 and 18^th^ June 2017 (when was at 12^th^ leaf stage) and harvested on 22^nd^ October 2016 and 23^rd^ October 2017. All plots were treated with basal fertilizer. Basal nitrogen (N) at 45 kg ha^−1^ as urea, phosphorus (P) at 40 kg ha^−1^ as calcium superphosphate, and potassium (K) at 150 kg ha^−1^ as potassium chloride were applied at the time of sowing in intercropped and sole-cropped maize. At the V6 stage of maize, the second dose of N was applied at 150 kg ha^−1^ as urea in all plots. The P at 60 kg ha^−1^ as calcium superphosphate, and K at 60 kg ha^−1^ as potassium chloride sulfate were basally applied for soybean at the time of soybean sowing. Other measures were used according to the farmer’s practices.

### Sampling and measurements

#### Light interception

**Fig 2:**
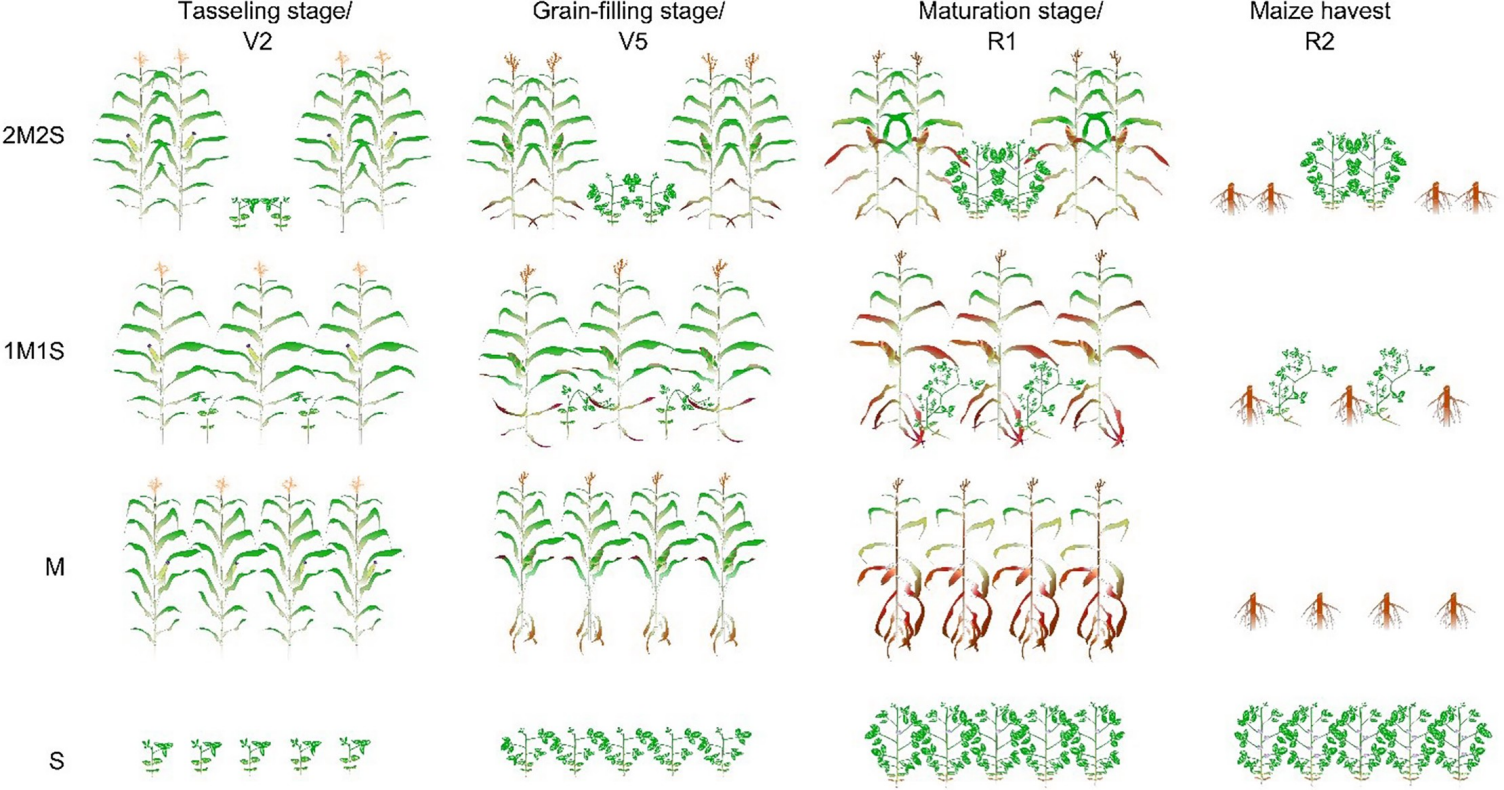
Schematic representation of different sampling stages of maize and soybean under different planting patterns of maize and soybean. The 2M2S (40 cm + 160 cm) and 1M1S (50 cm: 50 cm). The SM and SS refer to sole cropping system of maize and soybean, respectively. TS, GFS and MS refers to tasseling, grain filling and maturity stages of maize corresponding to second trifoliate (V_2_), fifth trifoliate (V_5_), flower initiation (R_1_) and full flowering after maize harvest (R_2_) stage of soybean, respectively.

For light interception measurement, photosynthetic photon flux density (PPFD) of all maize leaves in wide and narrow rows (from top to bottom) was measured at tasseling stage (TS), grain-filling stage (GFS) and maturation stage (MS). The PPFD of all odd (1, 3, 5, 7, 9, 11 and 13) and all even (2, 4, 6, 8 10 and 12) number leaves were measured in wide rows (WR; right side) and narrow rows (NR; left side), respectively under 2M2S, 1M1S and M. In addition, PPFD at the top of soybean canopy was also determined at second trifoliate stage (V_2_), fifth trifoliate stage (V_5_) and flower initiation (R_1_) stages corresponding to TS, GFS and MS stages of maize, respectively (**Fig 2**). The measurements were performed following a previously described method [28]. A color acetate film (O-1D, Taisei Chemical Industries, Tokyo, Japan) and Aquation Scientific Equipment (opto leaf, D-Meter RYO-470, Taisei Chemical Industries, Tokyo, Japan) were used to record and read the data. The film located at the middle of maize leaves for 1 day from 6:00 am to 20:00 pm for the collection of data. Six replicates were applied for the experiments and average was calculated. The PPFD was obtained as follows:

PPFDtotal(molm−2)=540.6−270.3×[log10(DD°)]×100

Where D_0_ is the initial light interception and D is the light interception after exposure.

#### Chlorophyll content

Six ear leaves (three from narrow and three from wide rows) of maize plants at TS, GFS and MS, and five fully expanded trifoliate of soybean plants at V_2_, V_5_, and R_1_, were collected from each treatment (**Fig 2**). The chlorophyll contents including Chl a, Chl b, and the ratio of Chl a/Chl b were extracted from all the leaf samples, and two leaf discs (1.130 cm^2^) were cut from the middle part of each middle lobules by a puncher (1.2 diameters), and dipped the samples in 10 ml of 80 percent aqueous acetone solution in the dark for 24 h at room temperature [29]. The extraction mixture was then measured at wavelengths of 663, 645 and 470 nm by using a spectrophotometer DU-730 (Beck Man Coulter Inc., USA).

#### Photosynthetic parameters

As described previously, the photosynthetic parameters of maize and soybean, including photosynthetic rate (*Pn*), transpiration rate (*Tr*), stomatal conductance (*Gs*), and intercellular CO_2_ concentration (*Ci*) were measured by using Li-6400 portable photosynthesis system (LI-COR Inc., Lincoln, NE, USA) under a CO_2_ concentration of 400 (μmol mol^−1^) [25]. In all treatments, six fully expanded ear leaves (three from narrow and three from wide rows) of maize leaves at TS, GFS, and MS, and five fully expanded soybean leaves at V_2_, V_5_, and R_1_ were selected (**Fig 2**), and the photosynthetic parameters were determined. The data collection of photosynthetic parameters was carried out from 10:00 to 12:00 h.

#### Morphological characteristics and leaf area

Ten maize and soybean plants were selected from each treatment to measure the leaf area at TS, GFS and MS, and V_2_, V_5_, and R_1_, respectively. Importantly, leaf area of all odd (1, 3, 5, 7, 9, 11 and 13) and all even (2, 4, 6, 8 10 and 12) maize leaves were measured separately in wide rows (WR; right side) and narrow rows (NR; left side), respectively. While plant height and stem diameter of maize and soybean were measured once at MS and R_1_, respectively and averaged was calculated. The plant height was measured from base to top and vernier caliper was used to measure stem diameter. In addition, the following equation was used to calculate the leaf area of maize and soybean [30]:

$$A_{t}({cm}^{2})=k(L\times W)$$

Where, A_t_ (cm^2^) represents leaf area of maize and soybean, L (cm) and W (cm) represent the maximum length and width values of maize and soybean leaves, for maize and soybean k was 0.7356 (R^2^ = 0.9553, p = 0.002) and 0.6903 (R^2^ = 0.9765, p = 0.001) in 2016 and 0.7298 (R^2^ = 0.9609, p = 0.001) and 0.6982 (R^2^ = 0.9732, p = 0.001) in 2017, respectively.

#### Biomass accumulation and distribution

Ten maize (five from narrow and five from wide rows) and soybean plants from each treatment were sampled destructively with at least one meter away from the last sampling at TS, GFS and MS, and V_2_, V_5_ and R_1_, of maize and soybean (**Fig 2**), respectively for biomass accumulation and distribution among different plant parts. Then all the sampled plants were divided into different plant parts of maize (leaves, stem and seed) and soybean (stem and leaves), and placed in oven for one hour at 65°C to kill the fresh-tissues and then dried at 80°C to obtain constant weight before weighing of each plant part of maize and soybean for total biomass accumulation (g plant^-1^) and distribution analysis.

#### Grain yield, land equivalent ratio and competition ratio

Furthermore, thirty-six ears (18 ears from narrow rows and 18 ears from wide rows) and forty soybean plants were sampled from the middle rows of each treatment at maturity. These samples were used to analyze the grain yield of maize and soybean. All the harvested sampled ears and soybean plants were sun-dried for six days, dried ears and pods were threshed by hand and weighed to measure the grain yield of every treatment and then converted into kg ha^−1^. Land equivalent ratio (LER) was also calculated by using the following equations [31,32].

$$LERm=\frac{LERim}{LERsm}$$

$$LERs=\frac{LERis}{LERss}$$

LER=LERm+LERs

Y_sm_ and Y_im_ are maize yields (kg ha^-1^) of sole cropping and intercropping system, respectively. Y_ss_ and Y_is_ are soybean yields (kg ha^-1^) of sole cropping and intercropping system, respectively. LERm and LERs are the partial land equivalent ratio of maize and soybean, respectively. LER more than 1 means that production in intercropping system is higher as compared to sole cropping system of its component species [31]. In addition, competition ratio (CR) is another parameter to investigate the competition between two crop species. The CR is determined by using the following formula:

$$CRm=\frac{LERm}{LERs}\times \frac{Zsr}{Zmr}$$

$$CRs=\frac{LERs}{LERm}\times \frac{Zmr}{Zsr}$$

Where LERm and LERs are the land equivalent ratio of maize and soybean respectively. Zsr and Zmr are the ratios of the area occupied by soybean and maize under the relay intercropping system relative to that of the corresponding monoculture, respectively (in this study, ratios of the area occupied by soybean and maize were the same) [32]. When the value of CR_m_ and CR_s_ is higher than ‘one’ suggested the competitive ability of maize and soybean greater than soybean and maize, respectively.

#### Economic analysis

To evaluate the economics of different planting patterns, an economic-analysis was conducted. Total expenditure for intercrops (maize and soybean) production was included farm-land rent, preparation of seedbed, seed and fertilizer cost of both intercrops (N, P and K), hand-weeding and thinning, harvesting and threshing of maize and soybean crops. Total income was calculated according to the local market price for maize and soybean at Chengdu in P. R. China in 2016 and 2017. Additionally, net income (NI) was measured by subtracting the total expenditure from total income and benefit to cost ratio (BCR) was assessed as the ratio of total income to total expenditure [25].

#### Data analysis

All parameters of planting models on the light environment, photosynthesis, chlorophyll content, biomass, and grain yield were analyzed using SPSS v17.0. Origin Pro 9.1 and Microsoft Excel program was employed to the graphical presentation of data. The least significance difference (LSD) test was used to compare the means at one percent or five percent probability level.

### Results

#### Light interception

**Fig 3:**
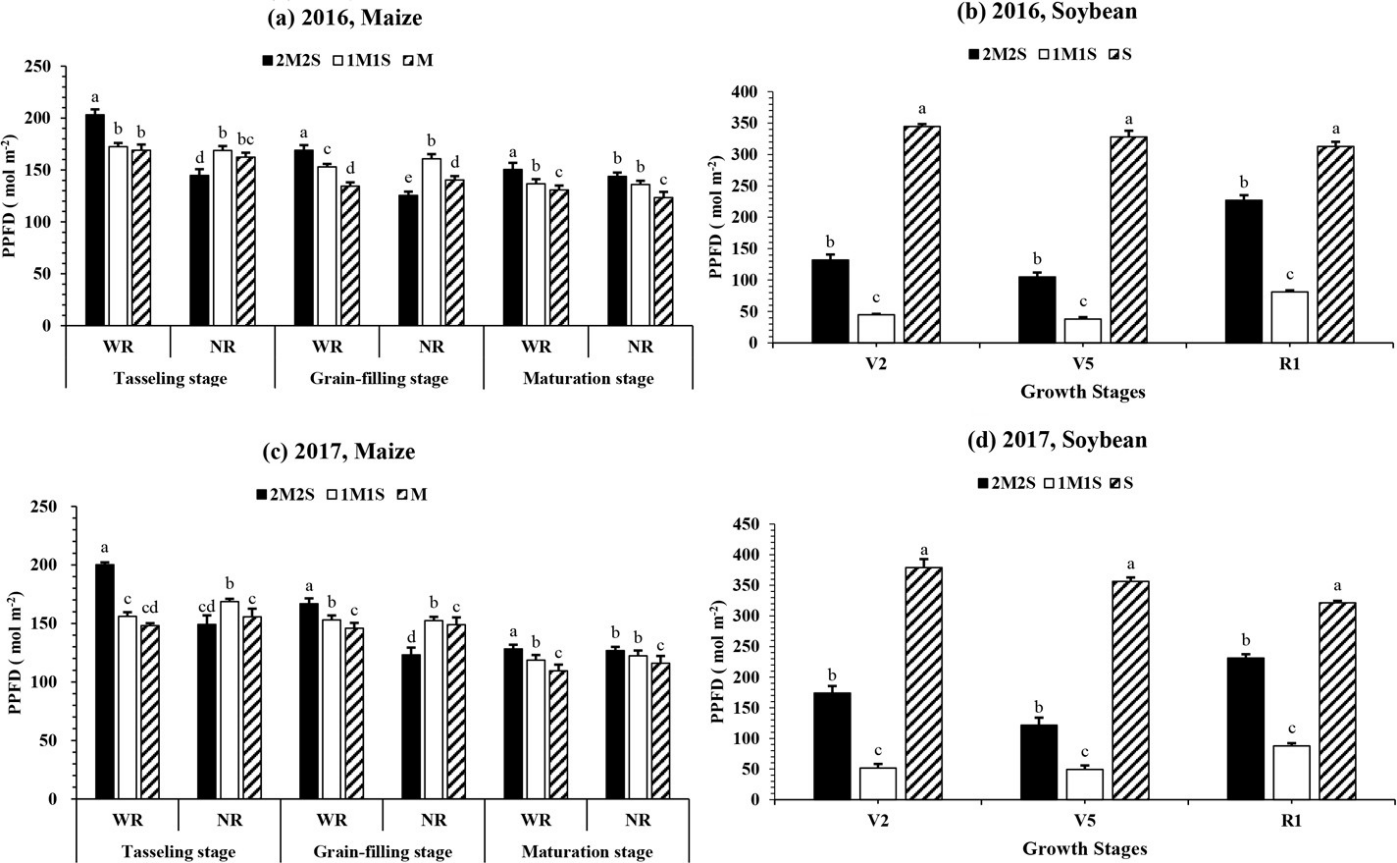
**The photosynthetic photon flux density (PPFD) of relay-intercropped maize (a, c) and soybean (b, d) at tasseling, grain filling and maturity stage of maize corresponding to second trifoliate (V**_**2**_**), fifth trifoliate (V**_**5**_**) and flower initiation (R**_**1**_**) stage of soybean, respectively as affected by different planting pattern from 2016 to 2017.** The 2M2S (40 cm + 160 cm) and 1M1S (50 cm: 50 cm). The SM and SS refer to sole cropping system of maize and soybean, respectively. The WR and NR wide rows (WR; right side) and narrow rows (NR; left side), respectively. Means are averaged over three replicates. Bars show ± standard errors, (n = 3). Within a bar, different lowercase and same letters show a significant and non-significant difference (*P < 0*.*05*) between treatments.

**Fig 3A and 3C** show the photosynthetic photon flux density (PPFD) at maize leaves at TS, GFS and MS under 2M2S, 1M1S and M. The different planting patterns treatments significantly (P < 0.05) affected the PPFD of maize leaves in wide rows (WR) and narrow rows (NR) at TS, GFS, and MS in both years. The mean highest PPFD of maize leaves was recorded in WR (right side; all odd leaves), and lowest PPFD of maize leaves was observed in NR (left side; all even leaves) at TS, GFS and MS, respectively for both years. Overall, the mean PPFD of maize leaves in WR under 2M2S was increased by 23, 10 and 9% in 1M1S and 27, 20 and 16% in M. In addition, we also calculated the total PPFD of whole maize plant (PPFD of odd leaves + PPFD of even leaves) and planting pattern 2M2S significantly increased the PPFD of whole maize plant at TS and MS by 5 and 7%, and 10 and 14% (**Table 2**), compared to 1M1S and M, suggesting that extra PPFD at odd leaves of maize in wide rows under 2M2S compensated the reduced PPFD effect at even leaves in narrow rows.

**Table 2:** Total photosynthetic photon flux density (PPFD) and light transmittance of relay-intercropped maize and soybean as affected by different planting pattern from 2016 to 2017.

Years	Treatments	Maize			Soybean		
		Total PPFD (mol m^-2^)			Light Transmittance (%)		
		TS	GFS	MS	V_2_	V_5_	R_1_
**2016**	**2M2S**	173.9a	170.6a	165.7a	38.3b	32.0b	72.7b
	**1M1S**	147.3b	156.8b	137.4b	13.0c	11.6c	25.9c
	**M**	147.1b	136.4c	127.1c	-	-	-
	**S**	-	-	-	100.0a	100.0a	100.0a
**2017**	**2M2S**	174.7a	162.3a	151.9a	45.9b	34.2b	72.0b
	**1M1S**	145.0b	152.7b	147.4b	13.6c	13.9c	27.3c
	**M**	127.5c	120.5c	112.7c	-	-	-
	**S**	-	-	-	100.0a	100.0a	100.0a

TS, GFS and MS refers to tasseling, grain filling and maturity stage of maize corresponding to second trifoliate (V_2_), fifth trifoliate (V_5_) and flower initiation (R_1_) stage of soybean, respectively. The 2M2S (40 cm: 60 cm: 40 cm: 60 cm) and 1M1S (50 cm: 50 cm). The SM and SS refer to sole cropping system of maize and soybean, respectively. Means are averaged over three replicates. Different lowercase letters in the same line are significantly different at 0.05 probability level.

The different planting treatments considerably (P < 0.05) changed the PPFD at soybean canopy in both two years (**Fig 3B and 3D**). However, the PPFD at soybean canopy in sole cropping system was always found higher than those under 2M2S and 1M1S at all sampling stages (V_2_, V_5_, and R_1_). In relay intercropping patterns, the average maximum and minimum PPFD at the top of soybean canopy were 153.0 and 48.2 mol m^2^ at V_2_, 113.3 and 43 mol m^2^ at V_5_, and 229.2 and 84.3 mol m^2^ at R_1_ under treatments 2M2S and 1M1S, respectively (**Table 2**).

#### Chlorophyll content

**Fig 4:**
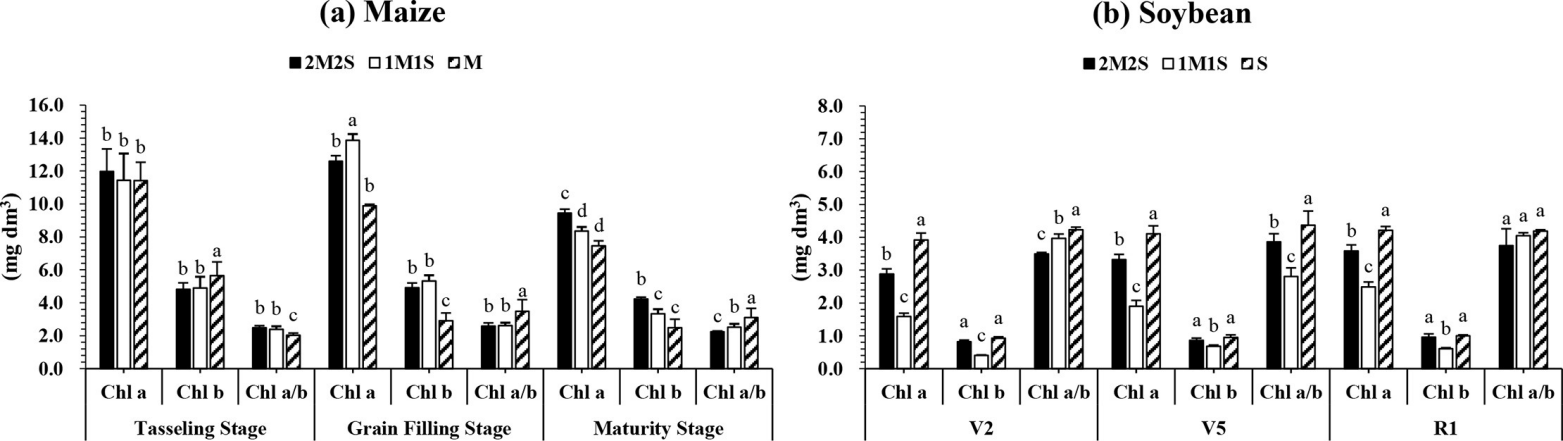
**The chlorophyll content of relay-intercropped maize (a) and soybean (b) at tasseling, grain filling and maturity stage of maize corresponding to second trifoliate (V**_**2**_**), fifth trifoliate (V**_**5**_**) and flower initiation (R**_**1**_**) stage of soybean, respectively as affected by different planting pattern averaged over 2016 and 2017.** The 2M2S (40 cm + 160 cm) and 1M1S (50 cm: 50 cm). The SM and SS refer to sole cropping system of maize and soybean, respectively. Means are averaged over three replicates. Bars show ± standard errors, (n = 3). Within a bar, different lowercase and same letters show a significant and non-significant difference (*P < 0*.*05*) between treatments.

In this study, different planting treatments considerably changed the contents of chlorophyll a and b in maize and soybean (**Fig 4A and 4B**). The Chl a content was significantly (P < 0.05) higher in maize under 2M2S at TS and MS as compared to 1M1S and M, while at GFS it was found maximum in 1M1S than 2M2S and M (**Fig 4A**). In addition, the Chl a and Chl b in soybean leaves under treatment S were increased significantly compared to those under 2M2S and 1M1S at V_2_, V_5_, and R_1_ stages of soybean (**Fig 4B**). Compared with 1M1S, the Chl a and Chl b contents were increased considerably by 81 and 106%, 75 and 27%, and 44 and 57% at V_2_, V_5_, and R_1_ under 2M2S, respectively (**Fig 4B**).

#### Photosynthetic rate

The photosynthetic characteristics of maize plants at TS, GFS, and MS under 2M2S, 1M1S and M are presented in **Table 3**. In our field experiment, different planting treatments significantly affected the photosynthetic rate of maize and soybean plants (P < 0.05). The mean maximum *Pn* of maize ear leaves was recorded in WR (right side) under 2M2S and in NR (left side) was measured in 1M1S at TS, GFS, and MS, respectively. The mean minimum *Pn* in WR, and in NR was noticed in M and 2M2S, respectively. Importantly, treatment 2M2S increased the *Pn* of maize ear leaves by 4 and 19% in WR and 21 and 40% in NR at MS than 1M1S and M (**Table 3**). The consistent pattern was observed for *Pn* of maize plants for 2016 and 2017 in WR and NR under different planting treatments. In addition, the values of *Tr* and *Gs* were found higher in wide rows than narrow rows under 2M2S as compared to 1M1S and M treatments for both years, while the *Ci* values were represented the opposite trend.

**Table 3:** Photosynthetic parameters of relay-intercropped maize and soybean as affected by different planting pattern from 2016 to 2017.

Years	Stages	Treatments	Photosynthetic Rate (μmol CO_2_ m^-2^ s^-1^)			Stomatal Conductance (mol H_2_O m^-2^ s^-1^)			Transpiration Rate (mmol H_2_O m^-2^ s^-1^)			Intercellular CO2 Concentration (μmol CO_2_ m^-2^ s^-1^)		
			Maize		Soybean	Maize		Soybean	Maize		Soybean	Maize		Soybean
			WR	NR		WR	NR		WR	NR		WR	NR	
**2016**	**TS—V**_**1**_	**2M2S**	28.8a	28.1b	9.7b	0.32a	0.29b	0.14a	2.93a	2.89a	3.3b	211.9b	216.5b	252.2b
		**1M1S**	28.5a	28.7a	5.5c	0.3a	0.31a	0.08b	2.91a	2.91a	2.5c	222.3b	229.2a	271.4a
		**M**	27.9b	28.2b	-	0.26b	0.27b	-	2.87b	2.83b	-	248.5a	236.4	-
		**S**	-	-	13.4a	-	-	0.16a	-	-	5.3a	-	-	205.2c
	**GFS—V**_**5**_	**2M2S**	26.8a	21.3c	11.6b	0.21a	0.11b	0.86b	3.51a	1.98c	3.9b	110.2b	176.3a	293.6c
		**1M1S**	24.2b	24.5a	5.6c	0.17b	0.15a	0.59c	3.26a	3.19a	3.5c	145.6a	142.8b	312.5a
		**M**	23.6c	23.5b	-	0.14b	0.14a	-	2.69b	2.64b	-	153.2a	156.8a	-
		**S**	-	-	14.8a	-	-	1.03a	-	-	4.3a	-	-	305.6b
	**MS—R**_**1**_	**2M2S**	19.3a	22.8a	12.9b	0.19a	0.20a	0.63b	2.88a	2.92a	3.8a	121.6c	119.8b	306.8a
		**1M1S**	18.6a	18.5b	8.8c	0.17a	0.15b	0.43c	2.76b	2.68b	3.4b	133.5b	135.2b	290.1b
		**M**	16.8b	17.1c	-	0.13b	0.13b	-	2.63c	2.58b	-	221.4a	226.5a	-
		**S**	-	-	14.5a	-	-	0.76a	-	-	3.9a	-	-	309.7a
**2017**	**TS—V**_**1**_	**2M2S**	26.6a	22.3c	9.3b	0.21a	0.19b	0.24b	2.69b	2.66b	4.1b	278.6a	289.4a	313.6b
		**1M1S**	24.1b	24.4a	6.1c	0.21a	0.22b	0.11c	2.72a	2.73a	3.5c	266.4b	253.1b	328.1a
		**M**	23.8c	23.8b	-	0.23a	0.24a	-	2.69b	2.68b	-	276.4a	273.5a	-
		**S**	-	-	13.1a	-	-	0.38a	-	-	4.6a	-	-	296.9c
	**GFS—V**_**5**_	**2M2S**	24.3a	20.8b	12.1b	0.18a	0.09b	0.93a	3.22a	1.67b	4.5b	148.5b	183.4a	318.9b
		**1M1S**	22.6b	22.4a	4.9c	0.13b	0.13a	0.62b	2.75b	2.73a	3.8c	162.8a	163.7b	333.2a
		**M**	22.1b	22.1a	-	0.13b	0.12a	-	2.69b	2.68a	-	163.1a	168.9b	-
		**S**	-	-	16.3a	-	-	1.03a	-	-	5.2a	-	-	320.5b
	**MS—R**_**1**_	**2M2S**	17.8a	21.3a	12.3b	0.15a	0.16a	0.86a	2.84a	2.84a	3.5b	158.2b	143.8b	286.9a
		**1M1S**	17.1a	17.8b	10.6c	0.14a	0.13b	0.25b	2.65b	2.66b	3.2c	162.8b	163.7b	273.3b
		**M**	14.5b	14.4c	-	0.11b	0.11b	-	2.45c	2.38c	-	256.8a	249.7a	-
		**S**	-	-	15.7a	-	-	0.93a	-	-	3.9a	-	-	292.5a

TS, GFS and MS refers to tasseling, grain filling and maturity stage of maize corresponding to second trifoliate (V_2_), fifth trifoliate (V_5_) and flower initiation (R_1_) stage of soybean, respectively. The 2M2S (40 cm: 60 cm: 40 cm: 60 cm) and 1M1S (50 cm: 50 cm). The SM and SS refer to sole cropping system of maize and soybean, respectively. The WR and NR wide rows (WR; right side) and narrow rows (NR; left side), respectively. Means are averaged over three replicates. Different lowercase letters in the same line are significantly different at 0.05 probability level.

The values of *Pn*, *Gs*, and *Tr* of soybean plants increased with the increase in PPFD at soybean canopy, and average values of *Pn*, *Gs* and *Tr* in treatment S at V_2_, V_5_ and R_1_ were found significantly higher than those under 2M2S and 1M1S for both years. However, planting pattern 2M2S significantly increased the mean *Pn* (by 64, 126 and 30%), *Gs* (by 100, 48 and 119%) and *Tr* (by 23, 15 and 12%) at V_2_, V_5_ and R_1_, respectively than 1M1S. Whereas, the maximum Ci was found in 1M1S and S at V_2_, and V_5_ and R_1_, respectively (**Table 3**).

#### Morphological parameters and leaf area

**Fig 5:**
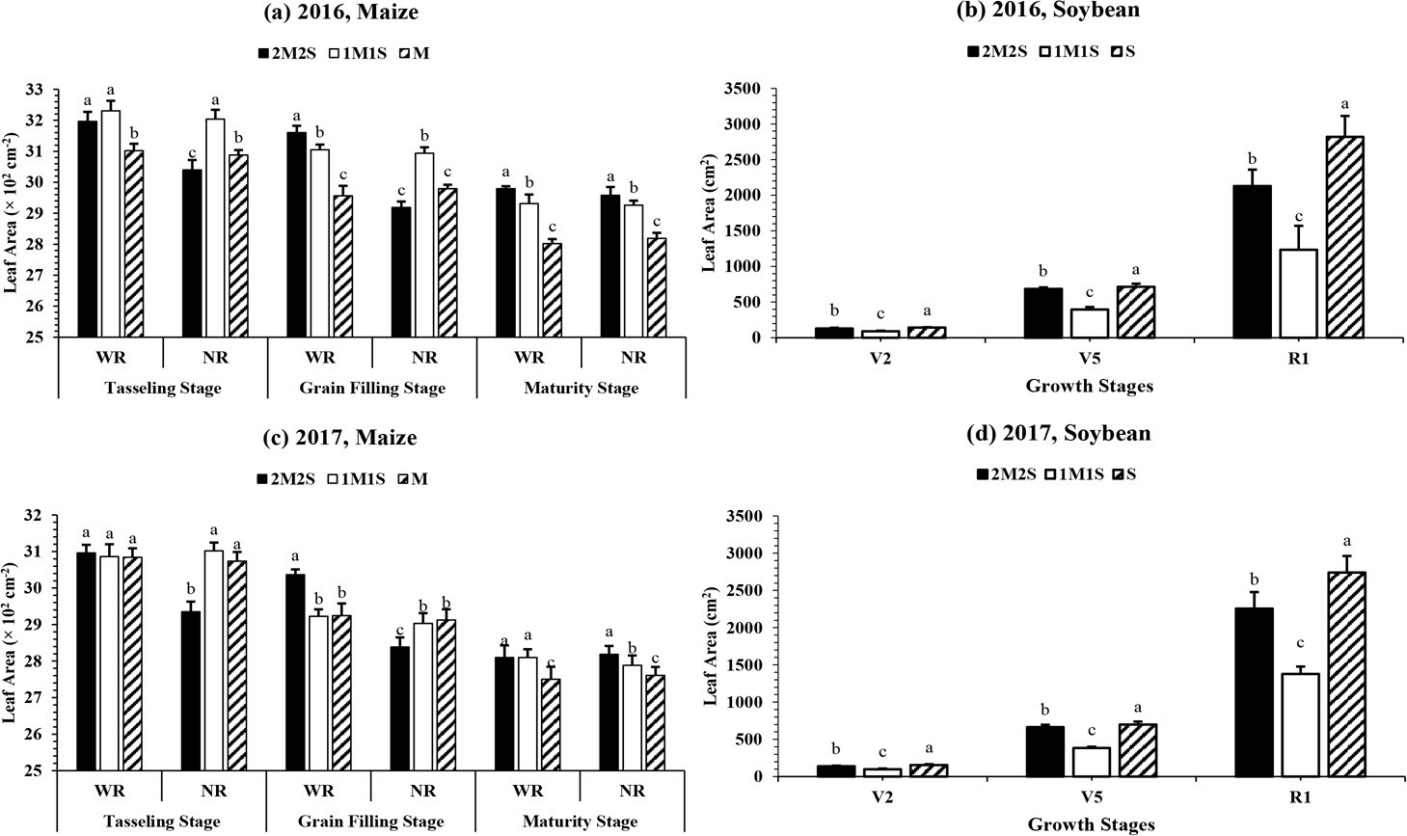
**The leaf area of relay-intercropped maize (a, c) and soybean (b, d) at tasseling, grain filling and maturity stage of maize corresponding to second trifoliate (V**_**2**_**), fifth trifoliate (V**_**5**_**) and flower initiation (R**_**1**_**) stage of soybean, respectively as affected by different planting pattern from 2016 to 2017.** The 2M2S (40 cm + 160 cm) and 1M1S (50 cm: 50 cm). The SM and SS refer to sole cropping system of maize and soybean, respectively. The WR and NR refer wide rows (WR; right side) and narrow rows (NR; left side), respectively. Means are averaged over three replicates. Bars show ± standard errors, (n = 3). Within a bar, different lowercase and same letters show a significant and non-significant difference (*P < 0*.*05*) between treatments.

In this study, different planting treatments significantly (P < 0.05) affected the morphological parameters and leaf area of maize (WR and NR of leaves) and soybean under 2M2S, 1M1S and M. During both study years, the plant height of maize plants in M were significantly higher than 2M2S and 1M1S (**Table 4**). However, the mean maximum stem diameter (26.1 cm) of maize plants was noticed under 1M1S as compared to 2M1S and M (**Table 4**). In addition, at MS, the mean maximum leaf area in WR and NR was recorded under 2M2S, whereas mean minimum leaf area was measured in M (**Fig 5A–5C**). Importantly, maize plants under 2M2S displayed the longer duration of green leaf area than M. For example, narrow-wide row planting pattern (2M2S) led to an increase in leaf area at MS by 4% in WR and 4% in NR, indicating that leaf senescence in 2M2S was delayed (**Fig 2**).

**Table 4:** Plant height, stem diameter and total biomass of relay-intercropped maize and soybean as affected by different planting pattern from 2016 to 2017.

Years	Treatments	Maize					Soybean				
		PH	SD	Total Biomass (g plant^-1^)			PH	SD	Total Biomass (g plant^-1^)		
		(cm)	(cm)	TS	GFS	MS	(cm)	(cm)	V_2_	V_5_	R_1_
**2016**	**2M2S**	262.8b	24.2b	127.8b	142.3c	247.7b	89.6b	8.6b	0.26b	3.57b	17.90b
	**1M1S**	260.9bc	25.9a	131.5a	145.9a	262.4a	110.3a	5.2c	0.20c	0.93c	7.68c
	**M**	273.1a	24.8b	130.0a	143.8b	263.7a	-	-	-	-	-
	**S**	-	-	-	-	-	84.2c	9.4a	0.42a	4.91a	35.66a
**2017**	**2M2S**	260.9b	23.8c	124.5b	130.9b	240.6b	91.2b	8.3b	0.27b	3.32b	17.52b
	**1M1S**	259.6b	26.2a	127.3a	135.7a	251.4a	106.8a	5.4c	0.21c	0.86c	5.52c
	**M**	272.8a	25.6b	127.5a	135.1a	250.2a	-	-	-	-	-
	**S**	-	-	-	-	-	84.6c	9.3c	0.46a	4.80a	31.94a

TS, GFS and MS refers to tasseling, grain filling and maturity stage of maize corresponding to second trifoliate (V_2_), fifth trifoliate (V_5_) and flower initiation (R_1_) stage of soybean, respectively. The 2M2S (40 cm: 60 cm: 40 cm: 60 cm) and 1M1S (50 cm: 50 cm). The SM and SS refer to sole cropping system of maize and soybean, respectively. Means are averaged over three replicates. Different lowercase letters in the same line are significantly different at 0.05 probability level.

All the planting treatments significantly (P < 0.05) affected the plant height (**Table 4**), stem diameter (**Table 4**) and leaf area (**Fig 5B–5D**) of soybean in both years. The mean maximum stem diameter and leaf area were observed in sole cropping of soybean. Whereas, the average highest plant height at all measured stages was recorded under 1M1S than 2M2S and S. However, treatment 2M2S increased the leaf area by 44, 72 and 68% at V_2_, V_5_, and R_1,_ respectively (**Fig 5B–5D**), and stem diameter by 59% (**Table 4**) compared to 1M1S at R_1_.

#### Biomass accumulation and distribution

**Fig 6:**
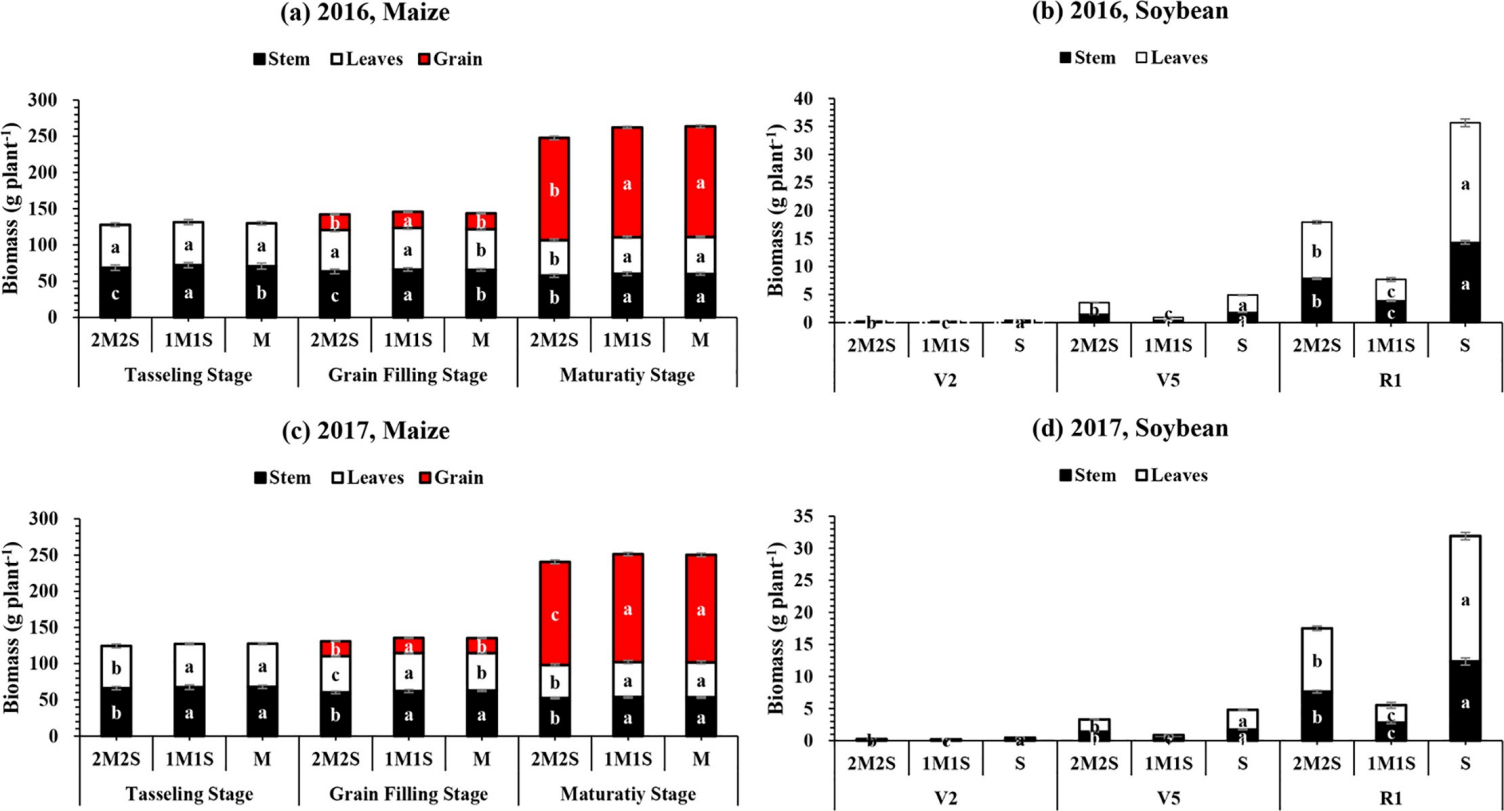
**The biomass distribution of relay-intercropped maize (a, c) and soybean (b, d) at tasseling, grain filling and maturity stage of maize corresponding to second trifoliate (V**_**2**_**), fifth trifoliate (V**_**5**_**) and flower initiation (R**_**1**_**) stage of soybean, respectively as affected by different planting pattern from 2016 to 2017.** The 2M2S (40 cm + 160 cm) and 1M1S (50 cm: 50 cm). The SM and SS refer to sole cropping system of maize and soybean, respectively. Means are averaged over three replicates. Bars show ± standard errors, (n = 3). Within a bar, different lowercase and same letters show a significant and non-significant difference (*P < 0*.*05*) between treatments.

The different planting treatments significantly (P < 0.05) affected the total biomass accumulation (g plant^-1^) in maize and soybean at all measured stages (**Table 4**). For maize plant, the mean highest biomass was found in 1M1S which was statically at par with M, while mean lowest biomass was recorded in 2M2S at all stages (TS, GFS and MS). For soybean, at R_1_, the mean maximum biomass 33.8 g plant^-1^ was noted under treatment S, while mean minimum biomass 6.6 g plant^-1^ was recorded under planting pattern treatment 1M1S, respectively. Furthermore, different planting treatments significantly changed the pattern of biomass distribution among different plant organs of maize and soybean (**Fig 6**). For maize, at TS and GFS, the maximum biomass allocation was observed in stem but after that (at MS) the highest biomass allocation was measured in seed followed by stem and leaves under 1M1S and M. On average, planting treatment 1M1S increased the seed biomass at MS by 8 and 5% in 2016 and 2017, respectively compared to the 2M2S (**Fig 6A–6C**). In addition, during all the measured stages, under S and 2M2S, highest distribution of biomass was recorded in leaves than stem, while in 1M1S maximum biomass distribution was noted in stem than leaves for both experimental years. Importantly, between 1M1S and 2M2S planting pattern treatments, the mean maximum leaf (9.93 g plant^-1^) and stem biomass (7.77 g plant^-1^) of soybean were found in 2M2S, while mean minimum (3.24 g plant^-1^) and stem biomass (3.36 g plant^-1^) of soybean were noticed at R_1_ under 1M1S (**Fig 6B–6D**).

#### Grain yield, land equivalent ratio and competition ratio

**Table 5** showed the seed yields, land equivalent ratio (LER) and competition ratio (CR) of maize and soybean under different planting treatments. The planting treatments significantly (P < 0.05) affected the maize and soybean seed yields, and LER under 2M2S, 1M1S. Higher seed yield of soybean (1642.1 in 2016 and 1702.2 kg ha^-1^ in 2017) was measured in 2M2S as compared to 1M1S (686.6 in 2016 and 707.1 kg ha^-1^ in 2017). On average, LER of 2M2S was increased by 27% and 31% in 2016 and 2017, respectively in comparison with 1M1S. Furthermore, the average maximum CR_m_ and CR_S_ values of maize and soybean were found in 1M1S and 2M2S, respectively, while minimum CR_m_ and CR_S_ values of maize and soybean were calculated under 2M2S and 1M1S, respectively in both study years.

**Table 5:** Seed yield (kg ha^-1^), land equivalent ratio (LER) and competition ratio (CR) of relay-intercropped maize and soybean as affected by different planting pattern from 2016 to 2017.

Years	Treatments	Maize			Soybean			LER
		Seed Yield	LERm	CRm	Seed Yield	LERs	CRs	
**2016**	**2M2S**	8472.3b	0.92b	1.25b	1642.1b	0.74a	0.80a	1.67a
	**1M1S**	9114.6a	0.99a	3.21a	686.6c	0.31b	0.31b	1.30b
	**M**	9168.7a	-	-	-	-	-	-
	**S**	-	-	-	2214.5a	-	-	-
**2017**	**2M2S**	8550.2b	0.95b	1.22b	1702.2b	0.78a	0.80a	1.73a
	**1M1S**	8952.5a	1.00a	3.07a	707.1c	0.32b	0.31b	1.32b
	**M**	8990.5a	-	-	-	-	-	-
	**S**	-	-	-	2181.8a	-	-	-

The LERm and CRm, and LERs and CRs represent the land equivalent ratio and competition ratio of maize and soybean, respectively. The 2M2S (40 cm: 60 cm: 40 cm: 60 cm) and 1M1S (50 cm: 50 cm). The SM and SS refer to sole cropping system of maize and soybean, respectively. Means are averaged over three replicates. Different lowercase letters in the same line are significantly different at 0.05 probability level.

#### Economic analysis

Results of the economic analysis are shown in **Table 6**. In this study, among different planting pattern treatments, 2M2S gave the highest net income (NI) (1387.7 US $ ha^-1^ for 2016 and 1434.4 US $ ha^-1^ for 2017), while average lowest NI (217.8 US $ ha^-1^ for 2016 and 188.7 US $ ha^-1^ for 2017) was obtained in S treatment. However, the mean maximum and minimum benefit to cost ratio (BCR) was measured with treatment M and 1M1S, respectively during both years. Overall, relay intercropping of maize and soybean with narrow wide row planting arrangement (2M2S) had 233% higher NI as compared to 1M1S.

**Table 6:** Economic analysis (US $ ha^-1^) for the effect of different planting patterns on maize and soybean performance pattern from 2016 to 2017.

Treatments	Total Expenses		Gross Income		Net Income		Benefit-Cost Ratio	
	2016	2017	2016	2017	2016	2017	2016	2017
**2M2S**	3339.9	3141.5	4482.4	4575.8	1341.0	1434.4	1.4	1.5
**1M1S**	3339.9	3141.5	3570.1	3544.5	428.6	403.1	1.1	1.1
**M**	1843.1	1733.6	2790.5	2736.2	1056.9	1002.6	1.6	1.6
**S**	1815.7	1707.8	1925.7	1896.5	217.8	188.7	1.1	1.1

The 2M2S (40 cm: 60 cm: 40 cm: 60 cm) and 1M1S (50 cm: 50 cm). The SM and SS refer to sole cropping system of maize and soybean, respectively.

## Discussion

### Effect of different planting treatments on light interception

The crop competition for sunlight is investigated in several studies about intercropping, for maize [33] and other annual crop species, such as soybean [8] and wheat [34,35]. In intercropping system, the planting system, row arrangement and spacing, and crop architecture can reduce the negative effects of taller crop shade on the middle strata leaves within the rows and between the rows. In our experiment, different planting systems considerably changed the light interception at maize leaves and soybean canopy, maximum light interception at odd and even leaves were observed in 2M2S and 1M1S, respectively (**Fig 3**). We noticed in another study of maize and soybean relay intercropping system that light interception and utilization was increased in 2M2S than 1M1S and S [12]. Furthermore, we observed, as Liu et al., (2017) did, the planting system 2M2S is favorable for higher maize and soybean seed yields because the increasing distance between maize strip was more advantageous to improve light interception at odd leaves of maize and soybean canopy, and past studies reported similar results [12,36,37]. Overall, wide rows in 2M2S increased the light interception at soybean canopy and compensate the decreased light intensity effect at even leaves of maize in narrow rows.

### Effect of different planting treatments on morphological parameters

Variations in light quantity can initiate crop morphological responses [38]. Generally, shading conditions under 2M2S and 1M1S significantly increased the plant height at the expense of leaves but it reduced the crop productivity [39,40]. Similarly, stem diameter of crops also reduced under low light conditions [41,42]. In our current experiment, minimum plant height, and maximum stem diameter and leaf area of soybean was observed in S (**Table 4**). However, between 2M2S and 1M1S, the higher leaf area of soybean plants was noticed under 2M2S as compared to 1M1S but the opposite results were found for maize leaf area (**Fig 5**). Furthermore, the leaf area of soybean is inversely proportional to shading [40], a decrease in light intensity reduces the leaf area of soybean by controlling the leaf proliferation under maize soybean relay intercropping system [22]. But narrow-wide row planting system significantly increased the leaf area of maize and delayed the leaf senescence process by increasing the leaf area at maturity (by 4% in WR and 4% in NR) in 2M2S. Therefore, our findings indicate that relay intercropping of maize and soybean in severe shading conditions under 1M1S probably promoted the stem elongation to obtain high amounts of light at the expense of leaf growth, which eventually reduced the crop growth and development of intercrop species. However, by using the narrow-wide row planting pattern (2M2S) we can grow soybean plants with higher stem diameter and leaf area, and it will be more beneficial to the initial growth of soybean plants under intercropping systems. Because increasing distance between maize and soybean rows under 2M2S reduced the maize shade, increased the light transmittance on soybean canopy, and decreased competition for land and water resources which eventually improved the maize and soybean growth.

### Effect of different planting treatments on photosynthetic characteristics

Under shading conditions, the investigation of chlorophyll content helps as an index for light absorption [21]. Several studies have documented that Chl a and Chl b contents decrease with the increase in shade [40,43]. On the other hand, other studies have argued that chlorophyll contents increase as shading density increases, especially Chl b content [16]. Our results showed that with the increase in the shade (1M1S) the Chl a and b contents were increased in maize leaves as compared to 2M2S and M in both years. In addition, the narrow-wide row planting treatments (2M2S) significantly increased the chlorophyll content of soybean leaves than 1M1S. This increase in chlorophyll content might be linked with the improved light environment and growing conditions for soybean plants under 2M2S (**Fig 4B**).

Plant leaves are responsive to the light conditions, and shading reduces photosynthetic capacity of crops [40,44]. This environment was consistent with our findings, which demonstrated that increasing maize narrow row distance (decreasing mutual-shading of leaves) enhanced ear-leaf photosynthesis at TS and GFS in 1M1S (**Table 3**) as compared to 2M2S and consequently significantly increased maize yield (**Table 5**). However, the net photosynthetic rate in wide rows under 2M2S was higher than those in 1M1S and M, which was the supplement (photosynthetic rate) for maize narrow row leaves. This increase in the photosynthetic rate of ear leaves in wide rows due to the improved light interception, leaf area and growing space for maize in 2M2S. Additionally, maize is a C_4_ and cereal crop that possess high photosynthetic and carbon gain activities [45]. By contrast, shading by relay-intercropped maize decreased the photosynthetic rate of soybean by reducing leaf area (**Fig 5**). The shading of relay-intercropped soybean became serious when the maize narrow row distance increased (1M1S, 50 cm: 50 cm). Whereas, the planting system 2M2S significantly improves the transmitted light at soybean canopy than 1M1S (**Table 2**). Therefore, these results indicated that the narrow wide planting pattern (2M2S) exhibited a higher photosynthetic rate of soybean than equal row planting system (1M1S) which increased the dry matter production and final seed yield of soybean plants by maintaining optimum maize yield.

### Effect of different planting treatments on intercrop yields, LER and CR

The remarkable increase in intercrop maize and soybean has been attributed mainly to the high use of inputs, which makes plant to use and intercept sunlight more efficiently [33]. By managing the planting density, row arrangement, and spacing, we can increase crop yield in relay intercropping system [11]. In this experiment, significant differences were noted in the biomass accumulation (**Fig 6**) and seed yield (**Table 5**) of maize and soybean in 2M2S, 1M1S, M, and S for both years in field conditions. These variations in biomass accumulation and yield are likely due to the differences in light interception and planting arrangements. Moreover, mutual shading of intercrop crops considerably changed the light interception [46] and any change in light interception directly affect the photosynthetic capacity (leaf area) of crops [40]. The narrow-wide row planting arrangement of maize and soybean under relay intercropping condition substantially increased the soybean yield as compared to equal row planting arrangement which was might be due to the higher light transmission at soybean canopy, improved leaf area and enhanced photosynthetic rate of soybean canopy in wide rows especially during the co-growth period because initial growth and development of crops is very important to obtain higher seed yield [25]. Furthermore, the decreased seed yield of individual intercrop can be counterbalanced by an increase in total grain seed yield on an annual basis [47]. For example, reducing 5.78% seed yield of relay-intercropped maize from 1M1S to 2M2S treatment increased the relay-intercropped soybean seed yield by 140% (**Table 4**).

Total LER values were always higher than one in both relay intercropping systems (**Table 5**), which exhibits the yield benefit of the relay-intercropping system over sole cropping systems (M and S) due to the better utilization of land and environmental resources for crops growth and development [27]. Particularly, the mean values of LER under 2M2S was 1.7, which means that 70% extra farmland will be needed by the sole cropping of maize and soybean to equal the seed yields of relay-intercropping systems, showing intercrops advantage of using resources as compared with sole crops [11]. Similarly, Liu et al., (2017) reported higher LER values 1.3–1.4 in narrow wide row intercropping system of maize and soybean [13], which suggesting that increasing the distance between maize and soybean rows (40 cm: 60 cm: 40 cm: 60 cm) under relay intercropping system improved the growing conditions (light environment) and decreased the competition especially for nutrients [48]. In addition, less distance (52 cm) between maize and soybean rows negatively affected the light interception at soybean canopy [8,49,50], therefore it is an effective method to ameliorate the negative effects of maize shade on soybean in maize soybean relay intercropping system, which resulted in higher intercrop seed yields and LER under relay-intercropping system.

The partial values of CR clearly showed maize as the dominant crop specie the under relay-intercropping system. Similarly, in previous investigations, it has been proved that the CR values of maize were always higher than soybean [10,27]. Moreover, higher competitive ability of maize crop to exploit and use available resources i.e. light, land, and water in association with soybean or groundnut or chickpea has been confirmed by other scientists [32,51,52]. Whereas, in pea-rye intercropping the partial values of CRp of legume (pea) were greater than cereal (rye), which was the different trend which we observed as cereal (maize) was more aggressive and competitive (higher CRm than CRs values) than legume (soybean) [32]. In this study, fertilizer and water were not the limiting factors in all planting patterns, but the row spacing and arrangement were the vital factors which may dominate and become more important for increasing the maize and soybean seed yields under maize sowing relay intercropping system.

Local farming communities only approve that new planting pattern or innovation which produces more profit with fewer expenses [25]. In this experiment, the economic analysis revealed that net income (higher profit) were obtained by using the narrow wide row planting pattern (2M2S, 40 cm: 60 cm: 40 cm: 60 cm) for maize and soybean production under relay-intercropping systems in both years than 1M1S (**Table 6**). Importantly, our results of the present study revealed that optimum light transmission and distribution at soybean canopy and in maize plants, respectively have significantly increased the total biomass accumulation and distribution towards the reproductive parts in maize and soybean plants under relay intercropping system and ultimately it can be a source of maximum profit to the farmer.

### Conclusion

In the present study, light interception and distribution patterns were evaluated by using different planting patterns. As compared to traditional planting pattern (1M1S, 50 cm: 50 cm), modern narrow-wide-row planting pattern (2M2S, 40 cm: 60 cm: 40 cm: 60 cm) greatly improved the light environment of maize and soybean plants. Our results indicate that greater contributions for relay-intercrop advantages can be attributed to better light interception and transmission between relay-intercropped species in maize-soybean relay intercropping system. Additionally, the high LER (1.7) and net income (1411.1 US $ ha^-1^) of 2M2S (40 cm: 60 cm: 40 cm: 60 cm) was a result of high maize and soybean seed yields (**Tables 5 and 6**). In relay intercropping with a enough distance between maize and soybean strips for growing soybeans, the improved light interception at maize narrow row and wide row leaves close to the ear increased their photosynthetic rate, and potentially maintained the maize biomass production and seed yield; the increased light at top of soybean plants significantly enhanced the light transmittance and photosynthetic rate of soybean, which then considerably improved its biomass production, competitive ability and seed yield. Therefore, advantage of relay intercropping can be improved by decreasing the competitive ability of maize.
